# Like Bits in a Jigsaw: Exploring Ancillary Staff Interactions With Care Home Residents

**DOI:** 10.1111/opn.70075

**Published:** 2026-04-15

**Authors:** Kritika Samsi, Olivia Luijnenburg, Caroline Norrie, Stephen Martineau, Ian Kessler, Emerita Jill Manthorpe

**Affiliations:** ^1^ NIHR Policy Research Unit in Health & Social Care Workforce King's College London London UK; ^2^ Professor of Public Policy and Management, King's Business School King's College London London UK

**Keywords:** ancillary workforce, care homes, qualitative methods, relationship‐based care

## Abstract

**Objectives:**

To explore accounts of the interactions between ancillary workers and care home residents during COVID‐19 pandemic lockdowns.

**Design:**

Qualitative interview study, following appropriate ethical approvals and procedures.

**Setting:**

Participants were recruited from care homes in England and through known networks. Interviews were conducted online or face‐to‐face when pandemic‐related contact restrictions were lifted, according to participants' convenience and comfort.

**Participants:**

Sixty‐three participants were interviewed: 38 ancillary workers (9 cleaners, 14 catering staff, 12 housekeeping staff and 3 ancillary staff supervisors), 8 care home managers, 5 human resource managers, 5 care home residents and 7 family members.

**Results:**

The meaningful nature of interactions between ancillary staff and care home residents was evident in participant interviews, acknowledged by family relatives, residents, care home managers and ancillary workers. Interactions with ancillary workers contributed to care practices, and many relationships were enhanced during COVID‐19. Participants felt these interactions contributed to the ‘jigsaw’ of the care home environment, and often positively impacted on staff well‐being. Management support to enable these interactions was important and could make relationship‐based care more meaningful.

**Conclusions:**

Interactions between ancillary workers and care home residents are important, can contribute to staff well‐being and resident quality of life by fostering relationship‐based care and promote the sharing of health and care information.

**Implication for Practice:**

Manager and supervisor recognition of this workforces 2019 contribution to relationship‐based care and to improve overall care home environments is needed, as well as staff development opportunities to enhance these relationships too.

## Summary

**What does this research add to existing knowledge in gerontology?:**

• Ancillary workers in care homes can form important relationships with care home residents; these came to light particularly during COVID‐19 lockdowns when care homes closed to visitors.
• Ancillary workers have the potential to share valuable information about a resident's health and well‐being.

**What are the implications of this new knowledge for nursing care for and with older adults?:**

• The contributions by ancillary workers to resident health and well‐being should be fostered and taken into account in practices and planning.
• The potentially close or regularity of relationships between ancillary workers and residents should be shared with and supported by family carers.
• Ancillary care home staff may wish to take on greater direct care work or progression; such career pathways could be encouraged.

**How could the findings be used to influence practice, education, research, and policy?:**

• Ancillary workers who work unseen or in the background in care homes but often provide social and emotional support and assistance to residents beyond their job roles may be interested in career progression and should be alerted to such opportunities.
• Wider recognition of ancillary staff's contributions to relationship‐based care enables such staff to feel appreciated and valued at work and could be more prominent in operationalising relationship‐based care.

## 1 Introduction

Ancillary workers in care homes can be overlooked. Within these settings, these are the people who do the cleaning, catering, housekeeping and laundry. Reasons for the general invisibility of ancillary staff may be that they often undertake low‐paid, low‐status, task‐oriented roles, shift‐patterned work; often out of sight of residents and visitors in kitchens, laundry areas or when undertaking the cleaning of often unoccupied areas. Their encounters with other professionals, family members and other visitors are likely to be brief but, by delivering discrete services, they make a significant contribution to residents' well‐being.

We report findings from a study investigating the contributions and experiences of ancillary staff working in English care homes during the Coronavirus pandemic. This pandemic (henceforth COVID‐19) highlighted some of the contributions of ancillary staff, with first‐person or other personal reports of their impact on care home residents' well‐being (Kale 2021; Wee et al. 2020; Luijnenburg et al. 2024) reflecting that of ancillary staff supporting hospital patients during that time (Wright 2021).

Wilson et al. (2009) identified three types of personal relationships extant in care homes: ‘pragmatic’ (that focus on instrumental aspects of providing care), ‘personal and responsive’ (that focus on individual needs of residents) and ‘reciprocal relationships’ (that recognise the contribution of residents, staff and family in creating a sense of community in a care home). This analysis of relationships builds upon the development of relationship‐based or relationship‐centred care developed by Nolan et al. (2001) to take further thinking around personhood or person‐centred dementia care beyond the focus on patient or care user. The contributions made, especially by cleaners, in everyday interactions with residents of other long‐term care facilities have been noted (Denton et al. 2004; Dancer et al. 2009; Richards 2005) and so reflect Wilson et al.'s typology but have not been explored in great depth. The personal and responsive or reciprocal relational aspects of such roles, in particular, are often overlooked, although one healthcare study identified how cleaning staff, possibly due to their role's apparent informality, enable seriously ill patients to express emotions about their condition (Jors et al. 2017). Likewise, in healthcare settings, reception or administrative staff were regarded as being important in a study of cancer patients experiencing pain (Schulman‐Green et al. 2005), and hospital porters in a study of patients with dementia were found to offer friendliness and reassurance (Ashton and Manthorpe 2019).

Using the term ‘hotel service staff’ to refer to staff working in long‐term Brain Injury Rehabilitation facilities in the United Kingdom, Latchem‐Hastings (2021) undertook a situational analysis of interviews and observations with 16 hotel service staff (undertaking catering, maintenance, cleaning and housekeeping) working in two such settings. She found that the caring acts and behaviours predominantly attributed to cleaners in other studies were also undertaken by the broader hotel service staff she studied, which, in this rehabilitation context, extended to involvement and encouragement of patients to be involved in daily living activities.

Turning specifically to care homes, an ethnographic study of cleaners working in care homes in six countries highlighted the relational aspect of their roles (Müller et al. 2018), through their carrying out of small but important acts of care and comfort. In their review of relationship‐centred care, Gurung and Chaudhury (2025), located three studies where participating care home staff held various roles, including dietary, housekeeping and administrative, and were reported to be initiating and sustaining supportive relationships with residents (Barken and Lowndes 2018; Marquis et al. 2004; Wilson et al. 2009).

### 1.1 Our Research Questions Were Therefore

What evidence from our study suggests that ancillary staff may be undertaking aspects of relationship‐based care? Was this a contextual pandemic‐related phenomenon or might this have lessons for the future?

We conducted a study exploring the ancillary workforce contribution during the pandemic in terms of infection control and management of contagion risks, and support available to them for their own well‐being in care home locations, many of which were experiencing substantial COVID‐19 outbreaks and sometimes deaths, often amid staffing pressures exacerbated by limited outside support from healthcare professionals (see Luijnenburg et al. 2024). This paper focuses on relationships between ancillary staff and care home residents as described by those we interviewed. It explores accounts of the experiences and contributions of a sample of the ancillary workforce in care homes in England, building on others' research in these settings during the pandemic (Nyashanu et al. 2020; Briggs et al. 2021). While some long‐term care facilities or care homes have a staffing model whereby staff who undertake personal care work also carry out domestic work such as cleaning, laundry and catering, in England, there is generally a role divide between staff offering personal care and those providing domestic or building‐based support. This is reflected in differentials in status, pay, training and development and career pathways.

## 2 Materials and Methods

### 2.1 Study Design and Ethical Considerations

This qualitative methods study was conducted during England's second wave of the COVID‐19 pandemic, from 1 January to 31 October 2021. It was funded by National Institute for Health & Care Research (NIHR) Policy Research Programme (NIHR202293).

Ethical approval was sought in two stages: King’s College London granted approval in February 2021 for interviews with care home managers, human resource (HR) managers and ancillary workers (ref HR/DP‐20/21‐21727). In May 2021, further ethical approval was received from Health Research Authority’s Social Care Research Ethics Committee for interviews with residents and relatives (ref 21/IEC08/0011). Our participant information sheet covered confidentiality and anonymity. Consent to participate was collected by the researcher prior to interviews. Efforts were made to minimise possible distress to participants when asked to recall difficult situations or times, and protocols were in place to address any safeguarding or other concerns that might emerge.

### 2.2 Data Recruitment

Using existing networks, social media, word of mouth and snowballing techniques, we recruited participants. The NIHR Clinical Research Network and the ENRICH network of care homes further helped publicise the study. All participating ancillary staff, residents and relatives were offered a £20 voucher in appreciation of their time. An Advisory Group (AG) of diverse stakeholders supported this study and aided recruitment by circulating study information to their networks. The research team met online with the AG before data collection in February 2021 to discuss overall approaches and questions, and in September 2021 to discuss emerging findings.

### 2.3 Data Collection

Data were collected between March and August 2021—between the second and third UK national lockdowns. Participants were interviewed at a mutually convenient time, using interview topic guides designed to ask ancillary staff about experiences of working in a care home during the pandemic, the role of ancillary staff during this time, and any support available for them. Interviews with HR managers included discussions about support offered to ancillary staff, while interviews with residents and relatives focused on their encounters with ancillary staff in the care home and the role they played in residents' daily lives. Interviews were conducted remotely, either over video‐conferencing facilities, such as Zoom or Teams, or via telephone and lasted between 25 and 65 min. All interviews with residents were carried out face‐to‐face in care homes when visitor restrictions were eased between lockdowns, and two interviews with relatives were also conducted in care homes face‐to‐face. Interviews were recorded, with permission, and brief demographic details were also collected. All recordings were transcribed and anonymised.

### 2.4 Data Analysis

Anonymised transcripts were analysed using reflexive thematic analysis (Braun and Clarke 2019), a process to scrutinise for themes and trends in the data. Line‐by‐line coding of transcripts was undertaken by OL and broad codes were identified. All participant transcripts were analysed together in order to identify overarching themes that were relevant to all groups. Between‐group differences were identified where they arose and not specifically sought. These were discussed with KS and organised into higher‐order codes, and a coding framework was iteratively developed as sub‐themes were identified. Consensus was reached through regular discussions with the wider research team, who were of mixed gender, age and ethnicity, had family caregiving experience, and included care home and business management academic experts. A ‘paper’ trail was maintained to ensure consistency and transparency; data analysis software NVivo (version 1.6.1) helped manage data.

## 3 Results

### 3.1 Participant Characteristics

Sixty‐three participants were interviewed: 38 ancillary staff, 8 care home managers and 5 HR managers, all of whom worked in care homes in England. Seven family members were also interviewed who had visited a relative living in a care home during this study's time period, and 5 residents. Of the ancillary workers, 9 were cleaners, 14 were catering or kitchen staff, 12 were housekeeping staff and three were hospitality or ancillary staff managers (see Table 1).

**TABLE 1:** Demographic details of 38 ancillary workforce participants.

Demographic category	Sub‐category	Total
Ethnicity	White British	23
	British Asian (Indian/Pakistani)	8
	British Asian Other (Filipino)	2
	Black African	2
	Black Caribbean	2
	Prefer not to say	1
Gender	Male	10
	Female	27
	Prefer not to say	1
Age (in year bands)	18–30	3
	31–40	7
	41–50	12
	51–60	13
	60+	3
Type of role	Cleaners	9
	Catering or kitchen staff	14
	Housekeepers	12
	Hospitality or ancillary staff managers	3

A core overarching concept from participant transcripts was the meaningful nature of interactions between ancillary staff and care home residents. This was acknowledged by family relatives, residents, care home managers and ancillary workers themselves. We delineated themes (see Table 2) that discuss how interactions contributed to relationship‐based care, how they were enhanced during COVID‐19, recognising these interactions as contributing to the ‘jigsaw’ of the care home environment, and the impact of this on residents and staff well‐being. We describe each theme, with associated sub‐themes, along with typifying participant quotes.

**TABLE 2:** Themes and sub‐themes: Interactions between ancillary staff and care home residents.

Themes	Sub‐themes
1. Contribution to care	1a. Building a sense of community
	1b. Holder of key resident knowledge
	1b. Involvement in care tasks
2. Commitment during COVID‐19	2a. ‘Home away from home’
	2b. Filling in for care work
	2c. Creativity and communication
3. Like bits in a jigsaw	3a. Contribution to homely environment
	3b. Care is more than just care work
4. Impact on staff well‐being	4a. Being a ‘people person’
	4b. Appreciating reciprocity of affection

### 3.2 Contribution to Care

#### 3.2.1 Building a Sense of Community

Despite not being at the frontline of delivering personal care, interviews highlighted that many ancillary staff knew residents well, their preferences and individual aspects of their character, with many relating accounts of affection in everyday interactions, all of which contributed to the sense of community in the care home:

*I go in slightly more often and there's sometimes a door open, the ancillary staff don't necessarily know I'm actually there. And one of them will be walking down the corridor, and they'll just stick their nose in and say, ‘Hi B, how are you darling, have you got everything you need?’ And to me it's been very personal. They do seem to care about her*. Relative 5

#### 3.2.2 Holder of Key Resident Knowledge

Participants described how information held by ancillary staff sometimes had the potential to influence care. For instance, a relative reported how the care home's cook realising that her relative's meal was being returned uneaten to the kitchen alerted her to a change in her mother's eating habits which precipitated a health review:

*I went in one day and [the senior cook] said “oh, can I have a quick word? I'm very worried. Your Mum seems not to be eating as much. I think she's struggling to eat. Do you think we can get somebody to see her so that maybe we can soften her food?” That was the cook! Not a carer, not the manager. So, I had Mum reviewed and she then was put onto a soft diet and her intake has improved*. Relative 5

Several managers reported that ancillary staff were likely to acquire information about residents in the course of their work. One recounted ‘if you want to know anything about our clients you go straight to our domestic or catering team’ (HR manager 2), and an ancillary staff supervisor reflected on the level of detail some of her staff possessed:

*The residents are known by their name and staff know their individual traits. [For] example, I said to [housekeeper] yesterday that a curtain needed attention in a room. And I said, “oh it's room number 9, it's Mr Y's room” and he said, “no, that's not Mr Y's room, Mr Y's room is number 8”*. Ancillary staff supervisor

#### 3.2.3 Involvement in Care Tasks

Participants also described examples of ancillary staff being involved in everyday care tasks that served the dual purpose of helping other care workers providing direct care, as well as contributing to residents' well‐being. Examples included cooking in the middle of the night if someone had been unwell and refusing their food during the day, as well as accommodating a new resident's food preferences:

*So, when we get a new resident in… normally I would introduce myself straightaway. I would ask about their likes and dislikes. I would try and gauge what sort of appetite they have… I like them to know my face, so if there's anything, they feel comfortable to ask me for it*. Chef 3

Many ancillary staff saw themselves as part of the fabric of the care home and valued their contribution to meeting residents' needs, with some demonstrating their knowledge of specific conditions and commitment to follow person‐centred principles:

*I was feeding [a resident] yesterday, slowly, I was considering that sometimes they can't swallow as quick as we can, and then I said, “wait, we have to wipe your mouth”. When I'm doing things, I let them know what I'm going to do next, I don't just jump in and start doing things, because I wouldn't want someone to do that to me, so why would I do that to a resident?* Cleaner 6

### 3.3 Commitment During COVID‐19

Managers often commented on their ancillary staff's greater interactions with residents during the COVID‐19 pandemic. Through enforced lockdowns, care homes stopped visitors, postponed communal activities and outings and residents were often unable to even have their usual social contact with one another. As a result, the contribution of the ancillary care workforce to the well‐being of residents, over and above their everyday tasks, was recognised and appreciated.

#### 3.3.1 ‘Home Away From Home’

Many participants reflected that when care homes were required to close their doors to visitors, staff and residents became more tightly knit and internally focused. This created an atmosphere of closeness in some care homes where staff became more of a ‘family’:

*I think, just – it became a home from home more because obviously, because of the pandemic, people have been coming to work and going home, coming to work, going home. It has been very limited, hasn't it, where people have been allowed to go. So, I think it actually built the relationships more within the home because of that*. Manager 2

#### 3.3.2 Filling in for Care Work

Limited visitors, both family but also professionals, during COVID‐19 restrictions meant that some care home staff took on new roles and responsibilities to support residents' well‐being, including some ancillary workers who had previously not taken part in social activities:

*It was a very scary, difficult time. Visitors had stopped, all the other staff who would come to the care home to entertain them stopped, we had to cancel our programmes, school visits stopped. We took on entertaining residents ourselves – playing bingo, we staff would sing for them, play games, chat with them more than we used to. We tried very hard, so they did not feel the loss of their family's visits too much. We chatted with them, didn't ask them direct questions about their family but found different things to ask them*. Cleaner 7

#### 3.3.3 Creativity and Communication

New interactions sometimes resulted in change to everyday care and routine activities, requiring creativity and care to comfort distressed, confused or potentially lonely residents:

*We wore (infection control) masks, which really upset and shocked some of our residents. They are used to seeing our faces, and our smiles. We would stand a distance from them and peek out behind our masks for a second to say “hello!” and reassure them that it was us, the staff they knew well. We would also sing songs behind our masks that we all knew or ones I knew they liked. Because we do know all our residents very well – some of them have lived with us for years*. Chef 3

### 3.4 Like Bits in a Jigsaw

One participant described the contributions of the relationships ancillary staff had with residents as part of the larger ‘jigsaw’ of care home life: *it's like bits in a jigsaw puzzle, isn't it? If you don't have them all, then it's not complete (Relative 6)* and felt these interactions impacted hugely on the care home's ambiance and environment. This feeling was also inherent in other participants' accounts.

#### 3.4.1 Contribution to Homely Environment

Managers, reflecting on the value of a homely environment, specifically said close relationships between ancillary staff and residents were encouraged regardless of the pandemic to create such an atmosphere:

*That's one thing we teach all our staff… if a resident talks to you, you engage with them, we expect everybody to engage with residents. Whether you're a domiciliary or an (administrative) staff member, I would expect some sort of humanity and engagement from all our staff. It's their home, and we work in their home*. Manager 3

#### 3.4.2 Care Is More Than Just Care Work

Among systemic changes following the pandemic that some managers wished to sustain, one was to encourage ancillary staff relationships with residents, facilitated by allocating staff to specific residents or blurring roles within the team:

*There were lots of positive changes to be honest with you. We've been able to make the housekeeping role more linked with care and vice versa. So, we've managed to, sort of, take a little bit of a step sideways where we don't just have housekeepers doing housekeeping work, it's carers doing care work and wellbeing [staff] doing wellbeing work. It makes you realise that, actually, care is more than just care work. We look at everybody—we're looking at residents being supported in their daily living, and daily living also includes cleaning*. Manager 2

Managers' recognition that ancillary staff contributed to resident well‐being also meant that they were keen to nurture these relationships and foster opportunities for these to flourish:

*One of [the residents] particularly loves when I come and visit him, he loves when I feed him, so if I'm [working] on the floor, I will always be scheduled to feed him, because he just likes my company*. Cleaner 6

### 3.5 Impact on Staff Well‐Being

Alongside close interactions and relationships between ancillary workers and residents contributing towards a better care environment, these impacted on staff well‐being. Ancillary staff participants reflected on these interactions as making them feel valued, their workplace seem more welcoming and their sense of self as being enhanced.

#### 3.5.1 Being a ‘People Person’

Several ancillary workers remarked how much they enjoyed working closely with residents and how their job was more than the tasks they undertook:

*You've got to be a people person as well. It's not a job where you can just go and clean. You've got to form a relationship with that client because say you're on ground floor for a week, which you can be sometimes, you're gonna be a week in that room with that person. You have to be able to hold a conversation and chat to them*. Cleaner 2

For some, their relationships with residents evoked memories of their older relatives:

*…it's not even about the cleaning for me anymore. I think there's a part of you that gets so in tune with the residents that you care for them like a grandparent. So, your relationship between the work and the residents, it changes, it's like you do things now because of the love, not because you need to get paid*. Cleaner 6

One participant suggested that even for non‐care roles, recruitment efforts should stress that this was a ‘people job’:

*I love talking to the residents… We do have a laugh so that's the main thing as well. To help them get through their day… Yeah, I think getting the right people into the job would be a good thing… you know it's an important job. It's actually a really important part of care homes because you've got to love people, you've got to be understanding of people, and you've got to actually love your work. Because if you don't love your work, you're not gonna do a good job*. Housekeeper 8

#### 3.5.2 Appreciating Reciprocity of Affection

A significant part of building and sustaining a relationship was acknowledgement by residents of the ancillary care worker, despite their disabilities and communication challenges. In one interview, a resident suggested that managers should ask residents about the quality of individual ancillary workers:

*We as residents can see how good they are. Do they really appreciate how good they are in the office?* Resident 4

Ancillary staff also mentioned the showing of affection through language, but also through non‐verbal gestures, which indicated the reciprocity of these relationships and the impact this had on resident and staff well‐being:

*And then there's another lady called […] that I sometimes help support and feed, and she's really good, and the other day she said, “I love you!” And was blowing me kisses, and I was like wow, that really touched my heart*. Cleaner 6

## 4 Discussion

### 4.1 Limitations and Strengths

This study is limited in having recruited between the second and third national lockdowns of the COVID‐19 pandemic (March 2021 onwards), when care homes were dealing with rapidly changing circumstances, consequently, recruitment was challenging. We may have only captured largely positive experiences, those with more negative accounts being too busy to engage with our research or feeling negatively about their experiences. Examples of poor experiences among staff such as cleaners have been reported (see https://www.workersstories.org.uk/2020‐6‐12‐my‐experience‐of‐being‐a‐cleaner‐in‐a‐care‐home‐through‐covid‐19/). We relied on participants' accounts of their work and interactions which may have contained biases related to the emotional context of the pandemic. However other studies using an ethnographic method prior to the pandemic have found similar relationship patterns (e.g., Müller et al. 2018).

The impact of the pandemic on many care home residents and their staff was profound globally (see Comas‐Herrera et al. 2022) as in England (Department of Health and Social Care 2023). This paper investigated the experiences and contributions made by cleaners, chefs/cooks, housekeepers and laundry staff working in care homes to relationship‐centred care. Whilst these roles share activities in common, many aspects of their roles differ (not least the tasks, training opportunities, pay and status of each). Evidently, intrinsic elements of the roles have implications for the scope of interactions between care home staff and residents; for example, cleaners and laundry staff were more likely to encounter residents in their bedrooms than other ancillary staff.

A strength of our study is that our sample contained care homes across England, with different staffing profiles and numbers of residents, which offered varied scope for reconfiguring the workforce and their tasks/roles and interactions during the pandemic. Participants were also demographically diverse in age, ethnicity and their job roles.

There are wider dimensions of our findings beyond simple (yet valued) recognition of ancillary workers in care homes. These include the potential for job redesign to provide great flexibility of job roles, as well as more meaningful work that will enhance general well‐being (see Parker and Jorritsma 2020). While there is emerging evidence in the potential for job redesign among care workers to take on further tasks and skills (see Duan et al. 2025), there could be specific attention to other care home employees and their career aspirations. Job redesign may be worth exploring in the context of the care home environment. While job crafting may be one approach to enhancing workplace roles (Holman et al. 2024), it is important to acknowledge that in English care home contexts, there are regulations covering practice relating to health and safety, staff roles and employment practices.

### 4.2 Contributions and Commitment

Findings from our study shed light on the significant, meaningful and regular interactions many ancillary workers have with care home residents, spanning the spectrum of the three types of personal relationships that may exist in care homes (Wilson et al. 2009) and reflective of the operationalising of relationship‐based care. Often these appeared to have gone unnoticed prior to the pandemic, lost in the hectic pace of care home life, but the impact and value of these on individual residents, family carers and the wider care environment were especially noticed and appreciated during the pandemic.

Nurturing social relationships in care settings is central to enhancing the well‐being of residents, family members and care staff Mjørud et al. 2017, if the value of each is recognised as a ‘partner of care’ (Kang et al. 2020); however, appreciating the potential contribution of ancillary staff in these interactions may further enhance the quality of the care environment. Ancillary workers may be uniquely placed to engage in some of these interactions, by nature of their work being around activities such as meal preparation and delivery, but they are not always engaged in helping with eating and drinking or promoting independence. These interactions enable them to acquire personal knowledge relevant to the care and well‐being of residents which otherwise might be overlooked.

### 4.3 Staff Well‐Being

Our findings support evidence indicating that positive relationships with residents contribute to staff well‐being and, in constituting a form of intrinsic reward, may assist in recruitment and retention (Awosoga et al. 2023), especially if enhanced by better terms and conditions of employment.

While the blurring of role boundaries in social care is prevalent (e.g., Abrams et al. 2019 in home care), some of this blurring may have been accentuated during the pandemic. For example, instances of a cleaner undertaking hairdressing for residents, a chef serving food or cleaning staff organising activities were reported by managers as having improved personalised care. Some of this blurring of boundaries could also be attributed to job crafting, where workers initiate behaviours to change their job characteristics, often with a view to changing the outcome for workers: in this case, their own well‐being (Holman et al. 2024). Whether these changes endure post‐pandemic needs to be ascertained, as well as determining whether this flexibility may help develop the career pathways of the ancillary care workforce. This approach may also reveal opportunities for the recruitment of cleaning and housekeeping staff into care roles, encouraging a ‘grow your own’ approach to care home recruitment. With training on specific conditions, such as dementia, potentially enhancing the quality of interactions with residents, ancillary workforces may be able to contribute more confidently to care home environments overall and resident well‐being (Haunch et al. 2021).

### 4.4 Management Support

Some studies predating the pandemic highlighted the contribution of the ancillary workforce to relationships in healthcare. Both domestic staff (cleaners) and hospital porters have reported substantial personal interactions with hospital patients living with dementia (Ashton and Manthorpe 2019). Another study found that cleaners working in care homes value the relational aspect of their roles (Müller et al. 2018), enacted through small but important acts of personal contact. However, other ancillary staff (e.g., catering and housekeeping workers) were not included in Muller et al. study; indeed, little has been researched about their contributions to the care home environment and to the relational aspect of their roles (Latchem‐Hastings 2021). Care home managers may wish to specifically recognise the commitment and contributions made by ancillary workers, many of whom in our study said this pre‐dated the pandemic. Support for care home managers in developing career pathways for their whole staff group may go some way towards enhancing relationship‐based care and research is needed on what model is most effective. In the context of future workforce challenges, creating well‐designed work that brings job satisfaction, personal fulfilment and enhances well‐being will be increasingly important to society (Parker and Jorritsma 2020). Overall, a wider societal shift may be needed to recognise that the cleaning industry can be more than just ‘mops and buckets’ (see https://www.cleaning‐matters.co.uk/page_1244998.asp) and that they and the wider ancillary workforce have a significant contribution to make to resident well‐being.

## 5 Conclusion

The quality of interactions in care homes is known to be important, and the concept of relationship‐based care reflects such thinking (Nolan et al. 2001), building on work recognising that there have been problems with the *‘*absent issue of the personhood of care staff’ more generally (Kadri et al. 2018). Recognising the role of ancillary workers in contributing to this and finding ways to support these workforces may improve overall care home environments, retention of staff and enhance both staff and resident well‐being. Manager support is needed to design workforce models that support these relationships and provide opportunities for these interactions beyond everyday task roles.

## Author Contributions

K.S. is the principal investigator of this study, designed and managed the project. K.S. analysed transcripts for this article. O.L. collected the data and undertook initial analysis. C.N. advised on ethics and undertook secondary analysis of the data. S.M. commented on developing analysis and earlier versions of this article. I.K. provided oversight and commented on versions of this article. E.J.M. provided mentorship throughout the study and provided detailed comments on this article.

## Funding

This work was supported by the National Institute for Health and Care Research, NIHR202293 and by the National Institute for Health Research Applied Research Collaboration South London.

## Conflicts of Interest

The authors declare no conflicts of interest.

## Data Availability

The data that support the findings of this study are available on request from the corresponding author. The data are not publicly available due to privacy or ethical restrictions.
